# Abortion, Anemia, and an Account of Idiopathic Intracranial Hypertension: A Case Report

**DOI:** 10.5811/cpcem.39958

**Published:** 2025-06-08

**Authors:** Chelsey Miller, Raphael Sherak

**Affiliations:** Yale University Department of Emergency Medicine, New Haven, Connecticut

**Keywords:** idiopathic intracranial hypertension, anemia, headache, abortion

## Abstract

**Introduction:**

Idiopathic intracranial hypertension is a rare but serious cause of headache. Prompt diagnosis and treatment is needed to prevent permanent neurologic sequelae.

**Case Report:**

We present a case of a 32-year-old female with multiple emergency department visits for a headache after having a medical termination of pregnancy. She was found to have severe anemia, retained products of conception, and radiographic findings suggestive of idiopathic intracranial hypertension, which was confirmed by an elevated opening pressure on lumbar puncture. Her symptoms improved after transfusion of packed red blood cells, initiation of acetazolamide and corticosteroids, and manual uterine evacuation. She was ultimately discharged without any neurologic deficits.

**Conclusion:**

Idiopathic intracranial hypertension is a rare but serious cause of headache, and anemia is an underappreciated precipitating factor. The hormonal changes associated with pregnancy may further predispose patients to this rare medical condition, especially in the setting of vaginal bleeding.

## INTRODUCTION

Idiopathic intracranial hypertension (IIH) is a rare cause of headache (1–2 cases per 100,000 people) that can cause disabling pain and the risk of severe vision loss.^1^,^2^ Given that it is a diagnosis of exclusion with a poorly understood pathogenesis, it often carries a prolonged diagnostic course. Below we present a case of IIH in the setting of anemia due to blood loss from a recent medical abortion.

## CASE REPORT

A 32-year-old female with a medical history of asthma, herpes simplex virus-2, and a body mass index (BMI) of 33 originally presented to the emergency department (ED) with a severe headache that had developed the day prior. She notably had no history of migraines, and her symptoms were refractory to home management with both ibuprofen and acetaminophen. She had no associated nausea, vomiting, or vision changes. Vital signs and neurologic exam were normal. The headache was thought to be a primary headache of benign etiology, and symptomatic treatment consisting of 15 milligrams (mg) of ketorolac, 10 mg of prochlorperazine, 975 mg of acetaminophen, and 1 liter of intravenous (IV) normal saline were ordered. Prior to administering the medications, a urine pregnancy test was ordered and resulted positive.

The patient subsequently disclosed she had had an elective termination of pregnancy with misoprostol 800 mg vaginally and mifepristone 200 mg orally two weeks prior to her presentation to the ED. A serum beta-human chorionic gonadotropin (β-hCG) resulted at 2,263 milli-international units per milliliter (mIU/mL) (reference range: <5 mIU/mL). She denied abdominal pain or vaginal bleeding. Her headache subsequently resolved after receiving the medications, and she was discharged to follow-up with primary care and obstetrics and gynecology (Ob/Gyn) for repeat β-hCG.

Eight days later, the patient re-presented to the ED complaining of an ongoing frontal headache with associated nausea since her previous visit, which had been refractory to at-home management. She again denied vision changes, vomiting, fevers, or neck pain. In the interim, she had experienced daily vaginal bleeding including passage of clots and had been using four to five sanitary pads a day. She had been in contact with her Ob/Gyn but had yet to schedule a repeat β-hCG and transvaginal ultrasound (TVUS). The patient was also on a waiting list for an outpatient neurology appointment. During this ED visit she again had a normal neurological exam, and her symptoms improved with a combination of ketorolac, metoclopramide, and an IV normal saline bolus. Her blood work during the visit consisted of a β-hCG of 488 mIU/mL, and point-of-care lab testing was normal aside from a hematocrit of 20% that was not addressed. After her symptoms remained improved, she was again discharged to follow-up with both Ob/Gyn and neurology.

Three days later, the patient had an outpatient ophthalmology visit where the ophthalmologist noted bilateral retinal hemorrhages and optic disc edema on exam. The patient was instructed to go directly to the ED due to concerns for elevated intracranial pressure (ICP). At triage the patient complained of ongoing headache, binocular blurred vision, and generalized weakness as well as persistent vaginal bleeding. Her vital signs were all within reference range for her age and sex, and her neurologic exam had no focal findings including fully intact visual fields. Her labs showed normal electrolytes, liver function, and thyroid tests but a hemoglobin of 5.9 grams per deciliter (g/dL) (11.7–15.5 g/dL) with a mean corpuscular volume of 93.8 femtoliters (fL) (80.0–100.0 fL). Her lethargy improved after a transfusion of one unit of packed red blood cells.

Neurology was promptly consulted, and she had a computed tomography (CT) angiography and computed tomography venography (CTV) of her head and neck as well as magnetic resonance imaging (MRI) of her brain that showed, *“Findings compatible with chronic/idiopathic intracranial hypertension, including partially empty sella, narrow transverse sinuses, prominence of the optic nerve sheath complexes, and bilateral papilledema”* (Image). No thromboembolism was found on imaging. The patient underwent a lumbar puncture (LP) that showed an opening pressure greater than 55 centimeters of water (cm H_2_O), and she had 20 mL of clear cerebral spinal fluid (CSF) removed with an improvement to a closing pressure of 19 cm H_2_O. Infectious testing was negative. The patient was not on retinoids, antibiotics, or oral contraceptives, and her only additional risk factor for IIH was an elevated BMI.

The patient was admitted to the neurology stepdown floor for a neurologic check every two hours and started on acetazolamide 500 mg twice daily for a presumptive diagnosis of IIH. On admission, she had a TVUS showing retained products of conception. Obstetrics/gynecology was promptly consulted, and she had a dilation and curettage performed on hospital day two. Following the procedure, she complained of ongoing headache, and she was treated with IV corticosteroids as well as migraine medications including metoclopramide and rizatriptan with good response. On the day of discharge (hospital day four), she reported a mild headache; however, visual acuity and fields remained normal. She was discharged home on acetazolamide, a solumedrol dose pack, and rizatriptan with plans to follow up with general neurology as well as neuro-ophthalmology.

*CPC-EM Capsule*

What do we already know about this clinical entity?

*Anemia can precipitate idiopathic intracranial hypertension (IIH). IIH is a rare cause of headache and if untreated can cause visual loss*.

What makes this presentation of disease reportable?

*This is a case of delayed diagnosis of retained products of conception causing severe anemia leading to IIH, highlighting a rare but critical linkage*.

What is the major learning point?

*Consider severe anemia as a risk factor for IIH in the differential diagnosis of headaches, especially in patients who are or recently were pregnant*.

How might this improve emergency medicine practice?

*Increased awareness of IIH, a time sensitive diagnosis, as a cause of headache in patients who were recently pregnant with anemia*.

## DISCUSSION

### Physiology and Diagnosis

Idiopathic intracranial hypertension is defined by the modified Dandy criteria: signs and symptoms of increased ICP (such as headache, vision loss, papilledema); normal neurologic exam; and an elevated ICP (>25 cm H_2_O on lumbar puncture) with normal CSF composition and no other primary cause of elevated ICP.^1^,^3^ The pathophysiology of IIH remains poorly understood but is speculated to involve increased CSF production via the choroid plexus and decreased CSF drainage through arachnoid granulations.^4^ Obesity is also a proposed risk factor attributed to increased production of leptin, 11β-hydroxysteroid dehydrogenase, or both, although the exact link is not fully understood.^1^,^2^

The relationship between severe anemia and IIH is controversial but hypothesized to involve a hyperviscous state leading to increased venous pressure. Another theory is that the reduced oxygen-carrying capacity of the blood results in cerebral hypoxia and edema precipitating headache, optic disc edema, and ultimately vision loss.^4^–^8^ It is curious that in the case presented above, the patient had significant papilledema and elevated ICP but no visual field deficits or changes to her visual acuity. Of the two cases presented in the literature of IIH following a medical abortion complicated by blood loss anemia, both had headache and an abnormal visual exam and only one had signs of elevated ICP.^9^,^10^ We were unable to find any evidence that misoprostol or mifepristone is associated with elevated ICP or IIH, and the association in this patient’s case is likely not causal. We believe it most likely that the vaginal bleeding from the incomplete medically assisted termination of pregnancy worsened the patient’s underlying anemia and subsequently precipitated fulminant IIH.

A thorough history and physical exam are crucial for diagnosing IIH. Headache is the most common symptom (over 80%), followed by visual changes (approximately 70%), dizziness, tinnitus, photophobia, and neck pain. Risk factors include pregnancy, rapid weight gain, and anemia as well as the use of contraceptives, fluoroquinolones, tetracycline, and vitamin A derivatives.^1^ When diagnosing IIH, a physical exam should include serial vital sign measurements, visual acuity and fundoscopic exam (or surrogate such as an ocular ultrasound for nerve sheath diameter), and comprehensive neurologic exam.^2^,^3^

The preferred imaging modality is MRI and magnetic resonance venography to assess for parenchymal lesion or meningeal process. In EDs where MRI is not readily available, second-line CT/CTV is acceptable particularly to rule out venous sinus thrombosis. As in our patient, neuroimaging may show signs of increased ICP such as empty sella, tortuous optic nerve, and enlarged nerve sheaths.^1^–^3^

To confirm a diagnosis of IIH, a lumbar puncture must be performed in the left lateral decubitus position. An opening pressure over 25 cm H_2_O is diagnostic.^1^–^3^ For significantly elevated pressures, clinicians should consider draining 20–30 mL of CSF and measuring the closing pressure. Just as in all routine LPs, glucose, protein, cell count, and cultures should be sent to rule out alternative etiologies.

### Management

The mainstay in IIH management is preserving vision along with alleviating symptoms. Both medical and surgical treatments exist (with varying degrees of efficacy) and highly depend on the severity of visual impairment. The first line of medical management for IIH is the use of 250–500 mg acetazolamide orally every 12 hours, a carbonic anhydrase inhibitor that decreases CSF production. This dose can be increased up to 4 g/day for severe vision loss or high-grade papilledema. Furosemide is an adjunctive medication that is sometimes used for long-term management, based on small-animal studies showing a marginal decrease in CSF production. Topiramate, also a carbonic anhydrase inhibitor, is another option for patients whose symptoms are refractory to acetazolamide as it has been shown to decrease CSF, but it also lacks robust data supporting its efficacy.^1^,^3^ For patients with proposed anemia-associated IIH, it is imperative that blood resuscitation occur. Given that this is an association without robust publication data, there is no consensus regarding target hemoglobin, but rapid resuscitation to “normal” values seems to improve outcomes.^4^

There are several surgical options for treatment of IIH: optic nerve fenestration; lumboperitoneal shunting; ventriculoperitoneal shunting; venous sinus stenting; and bariatric surgery. Serial LPs may be performed inpatient for patients with resistant symptoms but are not recommended on a routine basis. As was demonstrated in our case, medical and surgical therapies may abate symptoms, but often these patients continue to have headaches throughout their lives. Preventative measures such as weight loss, salt- and fluid-restriction dieting, correction of metabolic abnormalities (such as anemia), and discontinuation of any possible inciting medication are paramount.

## CONCLUSION

Idiopathic intracranial hypertension is a difficult diagnosis to make because it is a rare diagnosis of exclusion. However, clinicians should have a high index of suspicion for this diagnosis, particularly in overweight women of child-bearing age. While the incidence of IIH is rising and speculated to be due to the obesity epidemic, this is certainly not the only risk factor for the condition. Here we report a case of IIH associated with blood loss anemia and medical abortion. Anemia and pregnancy are established secondary causes of this disease, and more research is needed to understand the role that abortive agents such as misoprostol and mifepristone may play. A complete blood count should always be obtained if there is suspicion for IIH, even in the absence of an identifiable risk factor for anemia.

We can improve the outcomes of these patients by understanding the signs and symptoms of increased intracranial pressure, identifying which patients are most at risk, and performing thorough neuro-ophthalmologic examinations. Early diagnosis and interdisciplinary care with radiology, neurology, and ophthalmology to correct anemia and urgently lower ICP will prevent vision loss and result in the best outcomes for these patients.

## Figures and Tables

**Image:**
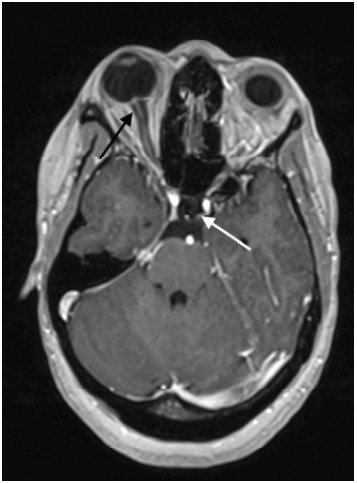
Axial T1 gadolinium enhanced magnetic resonance image of patient’s brain. Patient was found to have multiple findings consistent with idiopathic intracranial hypertension including partially empty sella (white arrow) and prominent optic nerve sheath and papilledema (black arrow).
